# Developing Conflict Monitoring Abilities Predict Children's Revision of an Intuitive Theory

**DOI:** 10.1111/cdev.14241

**Published:** 2025-04-01

**Authors:** Elfriede R. Holstein, Maria Theobald, Leonie S. Weindorf, Garvin Brod

**Affiliations:** ^1^ DIPF|Leibniz Institute for Research and Information in Education Frankfurt Germany; ^2^ IDeA‐Center for Research on Individual Development and Adaptive Education of Children at Risk Frankfurt Germany; ^3^ University of Trier Trier Germany; ^4^ Department of Psychology Goethe University Frankfurt Germany

**Keywords:** conflict monitoring, generating predictions, hypothesis testing, metacognition, violation of expectation

## Abstract

We investigated the role of children's conflict monitoring skills in revising an intuitive scientific theory. Children aged 5 to 9 (*N* = 177; 53% girls, data collected in Germany from 2019‐2023) completed computer‐based tasks on water displacement, a concept prone to misconceptions. Children predicted which of two objects would displace more water before receiving feedback. With increasing age, children showed slower response times for incorrect predictions (*β* = −0.04) and greater pupil dilation to unexpected outcomes (*β* = −0.04), indicating better conflict monitoring. Better conflict monitoring, in turn, predicted faster belief revision (*β* = 0.07). These findings suggest that conflict monitoring is crucial for learning in discovery‐based activities.

Science learning is an ideal setting to investigate how children revise intuitive theories. For example, consider children learning about what determines how much water an object displaces when fully submerged. As is true for many scientific phenomena, children have intuitive theories about what determines water displacement (Burbules and Linn 1988). A typical intuitive theory about water displacement is that an object's mass, or material, determines the amount of water displaced when fully immersed in water (Dawson and Rowell 1984; Linn and Eylon 2000; Piaget and Inhelder 1941). However, it is actually the volume, or size, of the object that determines how much water it displaces. Depending on their prior theories, learners generate different hypotheses. For example, when presented with a small, heavy object and a large, light object, a mass theorist would predict that the small, heavy object displaces more water, while a size theorist would predict that the large, light object displaces more water. Upon witnessing that it is actually the large, light object that displaces more water, mass theorists should use this new, conflicting evidence to revise their prior, intuitive theory (Colantonio et al. 2023a). This process reflects the child's active involvement in hypothesis testing, navigating the relationship between theory and evidence, as they build and refine their understanding of the world (Gopnik and Wellman 2012). Thus, learners have (intuitive) theories about the world that allow for hypothesis testing and theory revision.

The ability to test and revise intuitive theories is thought to develop early in life. According to recent versions of the “theory theory” of cognitive development (see Gopnik and Wellman 2012), children construct intuitive theories of the world starting in infancy (e.g., Gopnik and Wellman 1992; Smith et al. 1985). These intuitive theories involve coherent, abstract, and hierarchical representations of the world and allow one to generate hypotheses about future events, i.e., to predict what will happen next. In the face of conflicting evidence, theories change in a rational way in response to this evidence, reflecting an interplay between hypotheses and data. The “theory theory” also recognizes the variability and progressive nature of change in intuitive theories, which is grounded in the computational framework of Bayesian learning (Gopnik and Bonawitz 2015); learners progressively integrate prior knowledge with new evidence to update their beliefs in a principled, probabilistically coherent way. Hence, according to “theory theory”, children learn by constructing intuitive theories, testing hypotheses, and using new data to revise their theories in a rational, probabilistic way.

To benefit from hypothesis testing, learners must monitor whether an outcome is different from predicted. For example, when observing an experiment, learners have to compare the outcome of the experiment against their initially hypothesized or predicted outcome. This comparative process requires metacognitive skills, which involve monitoring one's understanding and self‐reflecting on one's current state of learning (Flavell 1979; Nelson and Narens 1990). However, learners often dismiss or rationalize conflicting evidence and persist in upholding their intuitive theories (Bonawitz et al. 2012; Chinn and Brewer 1993). Hence, learners often have difficulty even recognizing contradictory evidence (Limón and Carretero 1997; Theobald et al. 2024), especially when inconsistent information is presented more implicitly (Markman 1977, 1979). For example, Limón and Carretero (1997) found that younger students had more difficulty recognizing conflicts than older students; those students who recognized a conflict were more likely to revise their incorrect theory. Similarly, children who showed a stronger pupillary surprise response to incorrect predictions (an indirect measure of conflict monitoring) were more likely to revise their incorrect theory about water displacement (Colantonio et al. 2023b; Theobald and Brod 2021). However, these studies did not specifically focus on conflict monitoring or its development with age, nor did they include additional measures of conflict monitoring beyond pupil dilation. Revising beliefs in science requires conflict monitoring, a process that may be particularly challenging for children with lower metacognitive monitoring skills (Gunstone and Mitchell 2005). These earlier findings indicate that recognizing conflicts is important as it facilitates learning from hypothesis testing and, consequently, the revision of intuitive theories. However, what has remained unclear in previous research is the extent to which the ability to monitor conflict develops with age, and whether these improvements might facilitate the revision of an intuitive scientific theory.

Previous research suggests that the ability to monitor errors or conflicts continues to develop throughout childhood. Although metacognitive precursors of conflict monitoring emerge as early as infancy (see, e.g., Goupil and Kouider 2019, for a review), they continue to develop throughout childhood, particularly around age 6 when children enter formal schooling (Roebers 2014). It is also around age 6 that children begin to adjust their learning behavior to avoid errors (Davidson et al. 2006; Destan et al. 2014) and begin to benefit from corrective feedback (Carneiro et al. 2018). For example, in contrast to 5‐year‐olds, 6‐ and 7‐year‐olds devote more study time to difficult items compared to easy items (Destan et al. 2014) and slow their response times to avoid making errors (Davidson et al. 2006). Similarly, 6‐ and 7‐year‐olds, but not 5‐year‐olds, have been found to benefit from generating a guess followed by corrective feedback compared with passively studying information (Carneiro et al. 2018). These metacognitive monitoring and control processes are further refined during elementary school (Roebers 2014, 2017) and continue to develop well into adolescence (Schneider and Löffler 2016). In summary, children's ability to monitor unexpected outcomes and adjust their thinking and actions accordingly develops throughout childhood, often showing strong improvements around age 6. These findings suggest that children's ability to revise intuitive theories correlates with their development of conflict monitoring skills. However, research on the developmental progression in conflict monitoring ability and its relation to children's revision of intuitive theories is lacking. To fill this gap, the present study examined age‐correlated differences in the ability to monitor conflicts and revision of an intuitive scientific theory in a science learning task.

## 1 Measuring Conflict Monitoring

Asking children to verbally report encountered conflicts is challenging, especially at a young age. Such explicit measures necessitate self‐reports regarding confidence, uncertainty in responses, or assessments of one's own abilities or performance (Schraw 2009). However, this is challenging for children (see Roebers 2017; Schneider and Löffler 2016 for a review). For example, children's self‐reports often tend to be overly optimistic when assessing their current or future performance (Finn and Metcalfe 2014; Lipko et al. 2009). However, using more implicit measures, even younger children show signs of conflict monitoring. For instance, children show a longer response latency for incorrect as compared to correct responses, and longer response times correlate with diminished subjective confidence in an answer (Kälin and Roebers 2020; Koriat and Ackerman 2010). This hesitation and uncertainty before a likely incorrect response indicate the anticipation of a conflict, which is why we call it “pre‐conflict monitoring” here. Its characteristic features, that is, longer response times and lower confidence in an answer before committing an error, are already present in 2nd graders and become more pronounced with increasing age (Koriat and Ackerman 2010). In other words, even young children can anticipate conflicts, at least implicitly.

Another measure to assess children's conflict monitoring implicitly is pupil dilation responses. Physiologically, task‐evoked changes in pupil size are indicative of increased physiological arousal (Kloosterman et al. 2015; Preuschoff 2011), driven by the release of norepinephrine in the brainstem's locus coeruleus (Joshi et al. 2016). The locus coeruleus receives input from the anterior cingulate cortex (Aston‐Jones and Cohen 2005), which is activated by surprising events that violate prior expectations or when processing conflicting information (Alexander and Brown 2019; Ebitz and Platt 2015). For example, being confronted with an outcome that conflicts with one's expectation has been shown to reliably evoke a pupil dilation response (Breitwieser and Brod 2021; Krüger et al. 2020; Reisenzein et al. 2006; Theobald and Brod 2021), called the pupillary surprise response (Brod et al. 2018). This pupillary surprise response can be considered a physiological measure of children's conflict monitoring. It has recently been shown that children who received reflection prompts that facilitated conflict monitoring showed a greater pupil dilation response to unexpected outcomes (Theobald et al. 2024). Hence, the evidence from neurophysiological studies suggests that the magnitude of the pupil dilation response to unexpected outcomes can serve as a physiological indicator of enhanced conflict monitoring.

The pupillary surprise response also predicts learning from errors. The magnitude of the pupil dilation response following an error has been shown to predict a higher likelihood of being correct on the subsequent trial (Murphy et al. 2016; Theobald and Brod 2021). For example, in a science learning task, children who showed a larger pupil dilation response to an unexpected, theory‐incongruent trial were more likely to switch to the correct concept on the subsequent theory‐incongruent trial (Theobald and Brod 2021). In other words, those children who showed enhanced conflict monitoring were more likely to revise their intuitive theory. Thus, a greater pupil dilation response to incorrect predictions (as an index of conflict monitoring) may initiate control processes that support the revision of intuitive theories.

## 2 The Present Study

The goal of the present study was to examine the developmental relation between children's ability to monitor conflicts and their success in revising an intuitive scientific theory. For this purpose, we synthesized data from three previous studies in which children learned about water displacement. Children participated in a prediction‐with‐feedback task that consisted of a series of trials. On each trial, children predicted which of two objects would displace more water, or whether both would displace the same amount of water, before seeing the correct outcome.

Children's response times to make the prediction before seeing the outcome were assessed as a measure of children's pre‐conflict monitoring, indicating the anticipation of a potential conflict. Children's pupil size when seeing the correct outcome was recorded throughout the trials as measures of conflict monitoring. We hypothesized that children would show enhanced conflict monitoring before and after making an incorrect prediction with age, as indexed by longer response times to make a prediction for incorrect predictions and a greater pupil dilation response to incorrect predictions. Conflict monitoring after seeing the conflicting evidence should, in turn, help children learn from incorrect predictions, thereby facilitating the revision of their intuitive theory. We thus expected older children to show faster belief revision, as indicated by a steeper performance increase across trials, compared to younger children.

## 3 Methods

### 3.1 Participants

We used data from three studies that utilized the water displacement task. For the present analyses, we focus on data from children who participated in the prediction condition (details of the experimental conditions are provided below). In total, 214 five‐ to nine‐year‐old children participated in the prediction task. Of those, 177 made at least one incorrect prediction throughout the learning phase and could thus be included in the final sample (53% girls; *N* study 1 = 40; *N* study 2 = 85; *N* study 3 = 52). The mean age of the children was 7.02 years (SD = 1.06, range [5;9]). The mean age of participating children in study 1 (*M* = 8.03, SD = 0.70, range [7; 9]) was significantly higher than that of children in study 2 (*M* = 7.15, SD = 0.76, range [6; 9]; *b* = −0.87, *t* = −5.89, *p* < 0.001) and study 3 (*M* = 6.04, SD = 0.84, range [5; 7]; *b* = −1.98, *t* = −12.22, *p* < 0.001). All children were recruited and tested in a dimly lit room without external sunlight at a large natural history museum in Germany. Data collection took place in 2019 (Study 1), 2021 (Study 2), and 2022–2023 (Study 3). Parents gave written informed consent prior to testing, and ethical approval for all studies was obtained from the local ethics committee at the DIPF | Leibniz Institute for Research and Information in Education. The data and analysis code are openly available on OSF (https://osf.io/bzsx8/).

### 3.2 Design

In all three studies, children learned the concept of water displacement in an experiment with four phases: (1) a pretest without feedback that assessed children's initial concepts of water displacement, (2) a learning phase in which children answered a series of trials with feedback to acquire the correct concept, (3) a posttest that was similar to the pretest, and (4) a transfer test that tested whether children could apply their newly acquired concept to related tasks. Our main focus was to test children's conflict monitoring when they received feedback that conflicted with what they had predicted. Therefore, for the present study, we focus on data from the learning phase, where children received feedback on their predictions. Details of the pretest, posttest, and transfer test can be found in the corresponding papers of the three studies (see Theobald and Brod 2021; Theobald et al. 2024).

All three studies compared a prediction condition, in which children made predictions before seeing the correct outcome, with another experimental condition in a between‐subjects design. In Study 1, a prediction condition was compared with a postdiction condition in which children reported post hoc what they would have predicted after seeing the correct outcome. In Study 2, a prediction condition was compared to a prediction with reflection condition, which included additional reflection prompts that encouraged children to relate their answers to existing knowledge (“For each trial, think about how your answer relates to what you have already learned in the previous tasks.”). The reflection prompt was presented in the general task instructions and was not repeated throughout the learning phase. In Study 3, a prediction condition was compared to a condition in which children observed a fictitious child make a prediction that they only had to confirm. In the present study, we were interested in age‐related differences in how children monitor incorrect predictions. Therefore, we only used data from children in the prediction conditions; data from the children in the other conditions were not analyzed.

Next, we provide a detailed description of the general experimental procedure, stimuli, and learning phase design, which were mostly similar across the three studies. Discrepancies in procedures or design across studies are described in the respective sections.

### 3.3 General Procedure

Student assistants tested the children individually in a quiet adjoining room of the museum. Each experiment took approximately 35 min. Before the experiment, the student assistants demonstrated water displacement by pressing a polystyrene ball in a cup of water. It was emphasized that the object was intentionally held underwater to prevent the children from confusing the concept of water displacement with buoyancy. Furthermore, children were shown an object being submerged in water and were asked to explain what happened when the object was pressed and held under water. Once children provided a satisfactory response (e.g., “the water level rises when the ball is held under water”), the first task was started. We also made sure that the children knew all the materials (styrofoam, wood, lead). If a child did not know one of the materials, we showed them the material or explained what typical everyday objects are made of the material (e.g., a table is made of wood).

#### 3.3.1 Stimuli and Learning Phase Procedures

The learning phase comprised a computerized trial‐and‐error learning task (programmed in PsychoPy (Peirce et al. 2019)). During the learning phase, children completed a series of trials of a water displacement task. In each trial, children were presented with pairs of objects that varied in material and, thereby, mass (polystyrene, wood, lead) as well as in size (small, medium, large). For each trial, children predicted whether the left or right object displaced more water, or if both displaced an equal amount. Study 2 differed slightly from Studies 1 and 3 in the general task instructions. Here, children in the prediction condition were additionally instructed to indicate whether they expected the outcome, and children in the prediction with reflection condition were additionally instructed to relate their answers to what they had already learned. Predictions were assessed on a 5‐point‐scale (1 = “clearly left”, 2 = “maybe left”, 3 = “both objects displace an equal amount of water”, 4 = “maybe right”, 5 = “clearly right”). The numbers were printed on a horizontal button box. That is, the numbers on the rating scale corresponded to the physical location of the buttons and pictures shown on the screen, which facilitated intuitive use of the rating scale. After making their prediction, children received feedback. Children were shown a “+” under the object that displaced more water and a “‐” under the object that displaced less water. If both objects displaced an equal amount of water, a “+” was shown under both objects.

**FIGURE 1:**
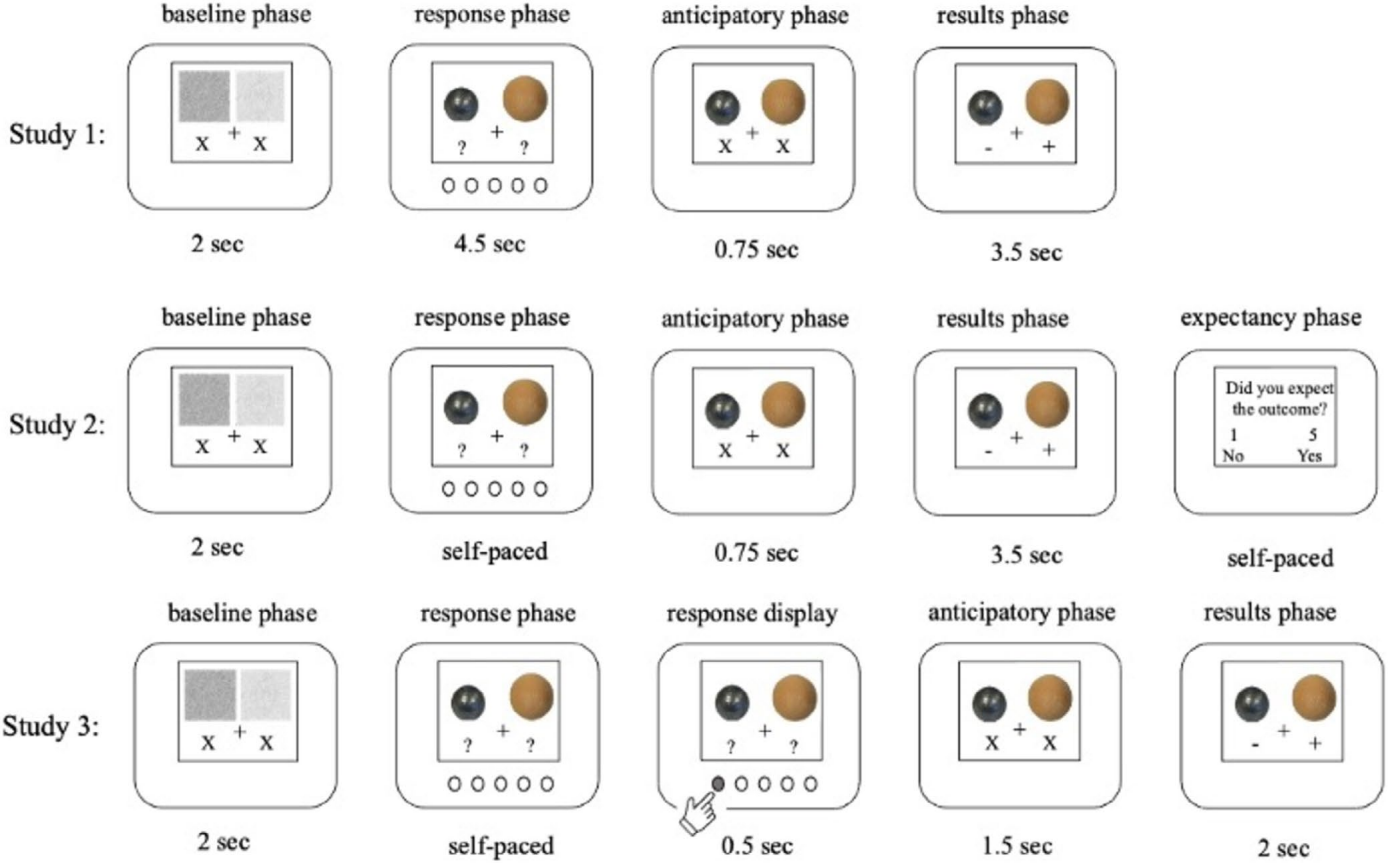
Trial sequence for Study 1, Study 2, and Study 3. All studies shared critical features; they had a ‘response phase’ in which predictions were generated, as well as an ‘anticipatory phase’ and a ‘results phase’ necessary to compute the pupillary surprise response. However, the exact sequence and timing of the trials differed slightly between the studies. In Study 1, children had a time limit of 4.5 s to make their predictions, whereas children in Studies 2 and 3 had no time limit. Unlike Studies 1 and 3, Study 2 included an additional ‘expectancy phase’ in which children were asked to indicate whether or not they expected the outcome. Unlike Studies 1 and 2, Study 3 had an additional ‘response display’ phase in which the prediction was shown. In addition, the ‘anticipatory phase’ was slightly longer and the ‘results phase’ was slightly shorter in Study 3 than in Studies 1 and 2.

The prediction conditions were very similar across the three studies. However, there were also small differences in the general task instruction, number of trials, stimuli, and trial sequence, which we describe below and in Figure 1. First, Study 2 differed slightly from Studies 1 and 3 in the general task instructions. In addition, Study 2 had a slightly different number of trials and used additional stimulus shapes (spheres, cones, and cubes vs. spheres only in Studies 1 and 3). There were also some temporal and content differences within the trial sequences between the studies (see Figure 1). In Study 1, children had a time limit to make their prediction. Study 2 included an additional expectancy phase at the end of the trial in which children indicated whether they expected the results or not. In Study 3, there was an additional response display phase, and the duration of the anticipation and result phases differed slightly from Studies 1 and 2. However, because rather long prediction, anticipation, and outcome phases were used in all studies, we were able to use the same time windows for our pupil and reaction time analyses across the three studies.

The experiment included (1) congruent trials in which the misconception (mass or material determines the amount of water displaced) led to the correct prediction (the heavier object was also the larger one), and (2) incongruent trials in which the misconception led to the wrong prediction. The three experiments had a varying number of congruent and incongruent trials. In Studies 1 and 3, the learning phase started with 8 congruent practice trials followed by a pseudorandomized sequence of 10 congruent and 16 incongruent trials, divided into two blocks. In Study 2, the learning phase started with 5 congruent practice trials followed by a pseudorandomized sequence of 7 congruent and 15 incongruent trials, divided into two blocks. In the practice trials, the larger object was always the heavier one, so these trials were not informative about whether water displacement depends on size or weight. An overview is shown in Figure 1.

#### 3.3.2 Eye Tracking Procedures

Using an eye‐tracking camera (EyeLink 1000, SR Research, Osgoode, Ontario, Canada), we assessed children's pupil size throughout the learning phase at a frequency of 500 Hz to measure the pupil dilation response to incorrect predictions. Each study began with a luminance‐matched ‘baseline phase’ at the start of each trial that was matched to the upcoming pictures of objects because pupil size is highly sensitive to changes in luminance. In addition, an ‘anticipatory phase’ was included before the ‘result phase’ to calculate pupil size changes in response to seeing the correct result. Furthermore, we ensured that the child fixated the center of the screen at the beginning of each trial by displaying a fixation cross in the center (see Figure 1).

### 3.4 Data Analyses

We used R (R Core Team 2019) for data analysis and set the significance levels at 0.05 throughout the analyses. The analyses tested the directional hypotheses formulated in the Introduction.

#### 3.4.1 Pupillary Data Preprocessing and Analysis

We used the same pupil preprocessing steps in each study. To prepare and analyze the pupil data, we used the ‘EyEdu’ Package (Korinth 2023). First, we identified periods of tracker loss, e.g., due to blinks or saccades, and interpolated missing values using fitted values. These fitted values were calculated by estimating a loess regression using 150 data points on either side of the regressed data point. We filtered data points with extreme variance in pupil size (SD > 750) and long blinks (> 100 ms) from each dataset (Study 1: 28%; Study 2: 29%; Study 3: 25%). Next, we epoched the pupil data relative to the onset of the “Results Phase”. We used the average pupil diameter during the last 300 ms of the “Pupil Baseline” of each trial as the pupil baseline measure. We then established a marker of surprise by calculating the change in pupil diameter 250–2000 ms after the onset of the “Results Phase” relative to each trial's pupil baseline.

#### 3.4.2 Implicit Measures of Conflict Monitoring

We conducted several analyses to test our hypothesis that children would show better metacognitive monitoring with age. First, we tested age‐related differences in the response times to make correct and incorrect predictions as a measure of children's pre‐conflict monitoring. PsychoPy automatically recorded the time (in seconds) it took the children to make their predictions. For this analysis, we excluded 42 data points where the children's response time deviated by more than 3 SD from the mean response time (1% of the data). We conducted a linear mixed effects model analysis (trials at level 1 nested within participants at level 2) to predict the response time to make the prediction at a trial‐by‐trial level. As predictors, we used age, prediction accuracy (1 = correctly predicted, 0 = incorrectly predicted), and their interaction.

Second, we tested whether the pupil dilation response is larger after incorrectly predicted compared to correctly predicted trials as a measure of the strength of conflict monitoring, and whether this relation differed by age group. Again, we calculated a linear mixed effects model with age, prediction accuracy, and their interaction as predictors of the pupil dilation response in each trial. Note that 10 children could not be included in this analysis as they had missing values on the pupil dilation measure for all incorrectly predicted trials. Moreover, for this analysis, we excluded 60 data points where the children's pupil dilation response deviated by more than 3 SD from the mean pupil dilation response (1% of the data).

Third, we tested whether a larger magnitude of the pupil dilation response to incorrectly predicted incongruent trials, as an index of enhanced conflict monitoring, predicted a higher likelihood of correctly responding to the next incongruent trial. To do this, we estimated a logistic mixed effects regression. We used pupil dilation response, prediction accuracy, trial type (0 = congruent trial, 1 = incongruent trial), and their respective interaction terms (assessed in a given trial *t*) as predictors of prediction accuracy in the subsequent trial (t + 1).

#### 3.4.3 Belief Revision During Learning Phase

To examine how belief revision evolves over trials, we used a logistic mixed effects model. Here, we tested the interaction between age and trial number as a predictor of performance throughout the learning phase. This analysis served to test the hypothesis that children would show a steeper increase in performance throughout the learning phase with age.

## 4 Results

### 4.1 Age Differences in Pre‐Conflict Monitoring

**FIGURE 2:**
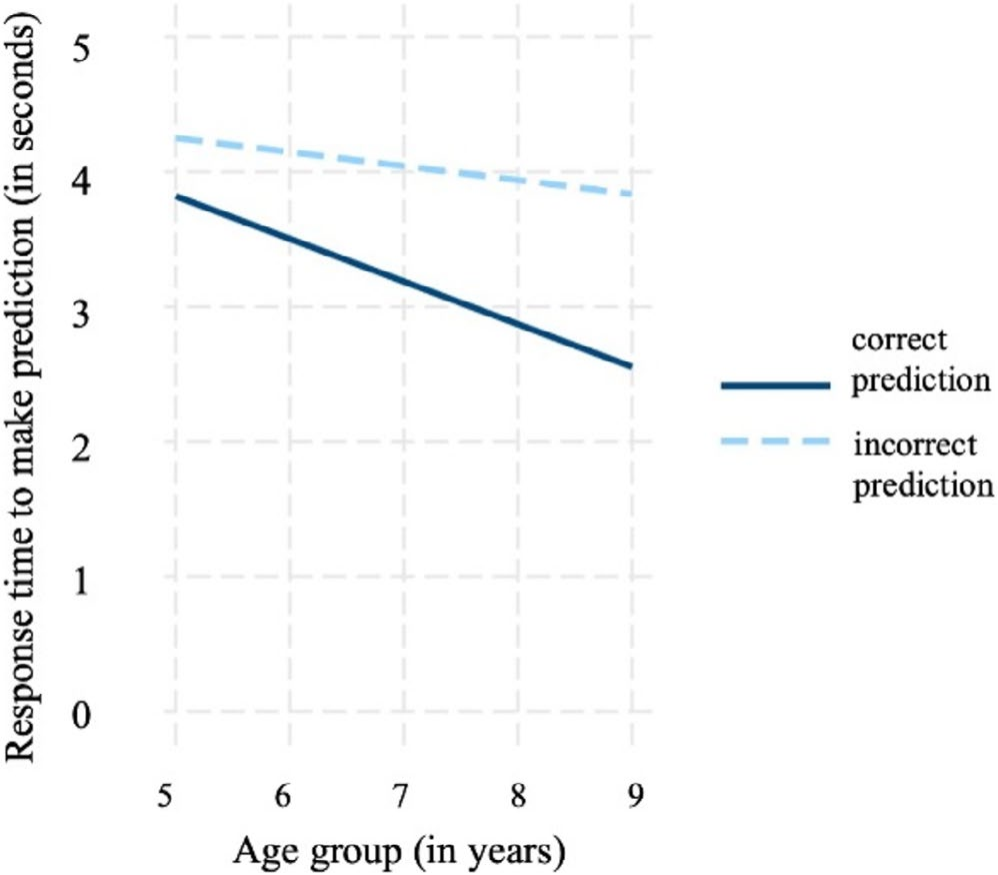
Response times (in seconds) for making correct predictions (solid line) and incorrect predictions (dotted line) divided by age group.

To examine children's pre‐conflict monitoring, we tested the hypothesis that children would take longer to make an incorrect (vs. correct) prediction. We found that children showed slower response times for incorrect compared to correct predictions (*b* = −0.86, standardized *β* = −0.15, SE = 0.08, *p* < 0.001, for details see Table S1, Model 1); this relation was additionally moderated by age (*b* = −0.21, standardized *β* = −0.04, SE = 0.08, *p* = 0.006, for details see Table S1, Model 2). Thus, slower response times for incorrect compared to correct predictions become more pronounced with age (Figure 2).

As a robustness check, we repeated the analysis with participants from Studies 2 and 3 only. Unlike Study 1, where children had a 4.5‐s time limit for predictions, children in Study 2 and Study 3 had no time limit and took significantly longer to make predictions (*b* = 1.82, standardized *β* = 0.35, SE = 0.18, *p* < 0.001). When excluding children from Study 1, we still find slower response times for incorrect (vs. correct) predictions (*b* = −0.85, standardized *β* = −0.15, SE = 0.08, *p* < 0.001), and that this effect is moderated by age (*b* = −0.20, standardized *β* = −0.04, SE = 0.08, *p* = 0.012). Thus, taken together, the slower response times for an incorrect prediction (relative to a correct prediction) can be seen as an implicit measure of children's pre‐conflict monitoring prior to actually seeing the correct outcome.

### 4.2 Age Differences in Conflict Monitoring

Second, we tested whether children show a stronger pupillary dilation response to incorrectly predicted trials. Consistent with previous findings (e.g., Theobald and Brod 2021), incorrect predictions elicited a stronger pupil dilation response compared to correct predictions (*b* = −0.77, standardized *β* = −0.07, SE = 0.18, *p* < 0.001, for details see Table S2, Model 1). These results suggest that unexpected outcomes elicited a pupillary surprise response.

**FIGURE 3:**
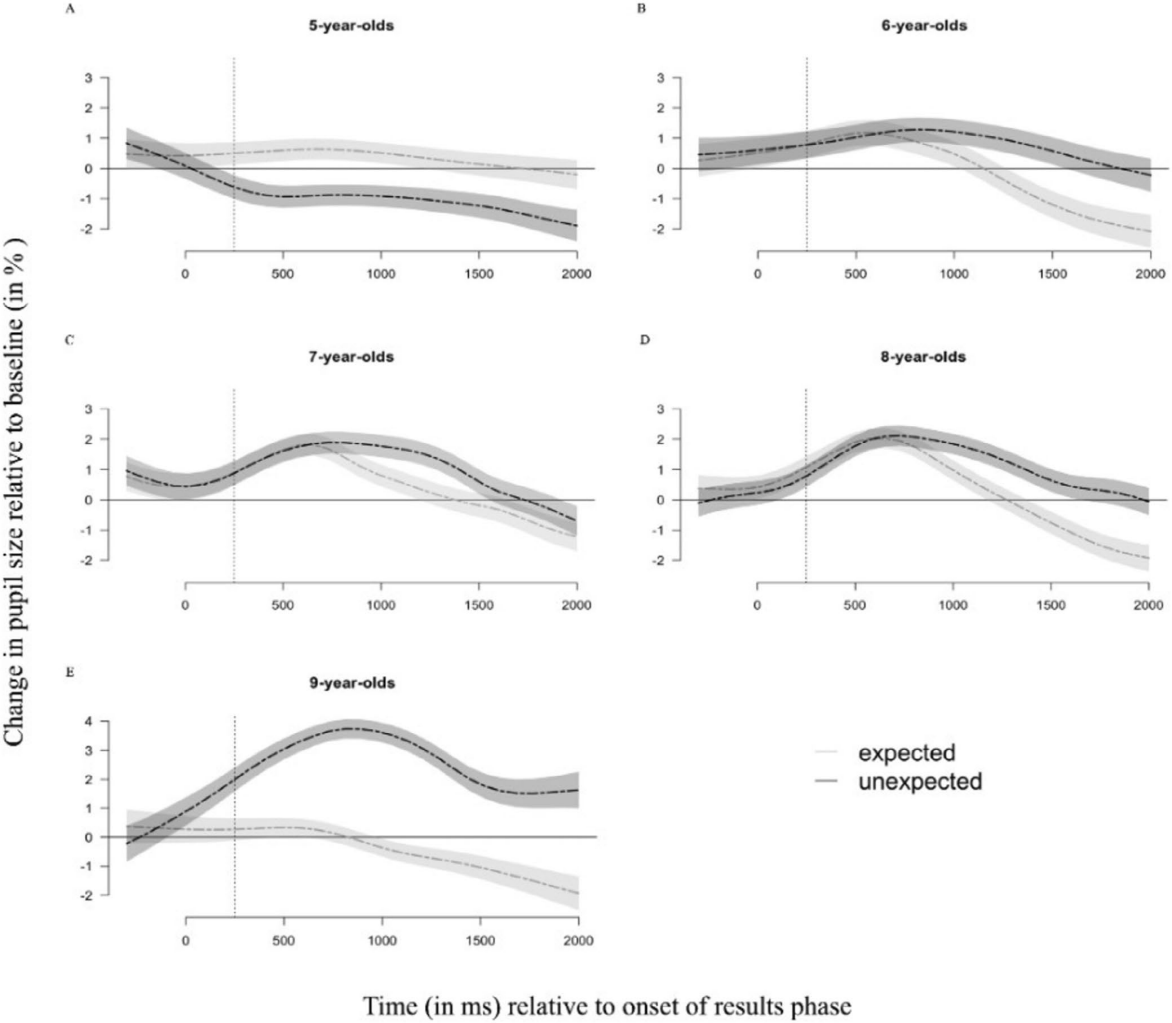
Pupil dilation response to correctly predicted (black line) and incorrectly predicted (gray line) trials by age group. The figure shows the pupil dilation response during the results phase when feedback on the correct answer was given for (A) 5‐year‐olds, (B) 6‐year‐olds, (C) 7‐year‐olds, (D) 8‐year‐olds, and (E) 9‐year‐olds. Shading indicates 95% CI. The pupil dilation response was computed over the time window from 250 ms to 2000 ms after the onset of the results phase; the dashed vertical line indicates the onset of this time window of interest. Plots of the pupil dilation response were generated after accounting for gaze position and autocorrelation of the residuals using a generalized additive mixed model (Van Rij et al. 2019; see Appendix B for more details).

Next, we tested whether age moderated the pupil dilation response after incorrectly predicted trials. Prediction accuracy and age interactively predicted the pupil dilation response (*b* = −0.38, standardized *β* = −0.04, SE = 0.18, *p* = 0.030, for details see Table S2, Model 2). Figure 3 shows the pupil dilation response to incorrectly predicted trials (vs. correctly predicted trials) for each age group. Examining each age group separately, we find that the pupil dilation response to incorrectly predicted trials (vs. correctly predicted trials) is present in 7‐year‐olds (*b* = −0.69, standardized *β* = −0.06, SE = 0.31, *p* = 0.027), 8‐year‐olds (*b* = −1.04, standardized *β* = −0.09, SE = 0.37, *p* = 0.005) and 9‐year‐olds (*b* = −1.96, standardized *β* = −0.18, SE = 0.71, *p* = 0.007), but not in 5‐year‐olds (*b* = 0.09, standardized *β* = < 0.01, SE = 0.50, *p* = 0.860) and 6‐year‐olds (*b* = −0.66, standardized *β* = −0.06, SE = 0.41, *p* = 0.106). Overall, these results suggest that the pupillary surprise response to incorrect (vs. correct) predictions became stronger with age, starting at around age 7.

We also examined whether children differed in the total number of errors they made during the learning phase. For example, younger children may have used a trial‐and‐error strategy in which errors are frequent and thus less surprising. In contrast, older children may have made fewer errors, which may elicit a stronger pupil dilation response. However, the number of errors was comparable across age groups, and age did not predict the number of errors made during the learning phase (*b* = −0.53, SE = 0.33, *p* = 0.118). This result rules out error frequency as an alternative explanation for the difference in pupil dilation response to unexpected outcomes between age groups.

### 4.3 Better Conflict Monitoring Predicts Belief Revision

**FIGURE 4:**
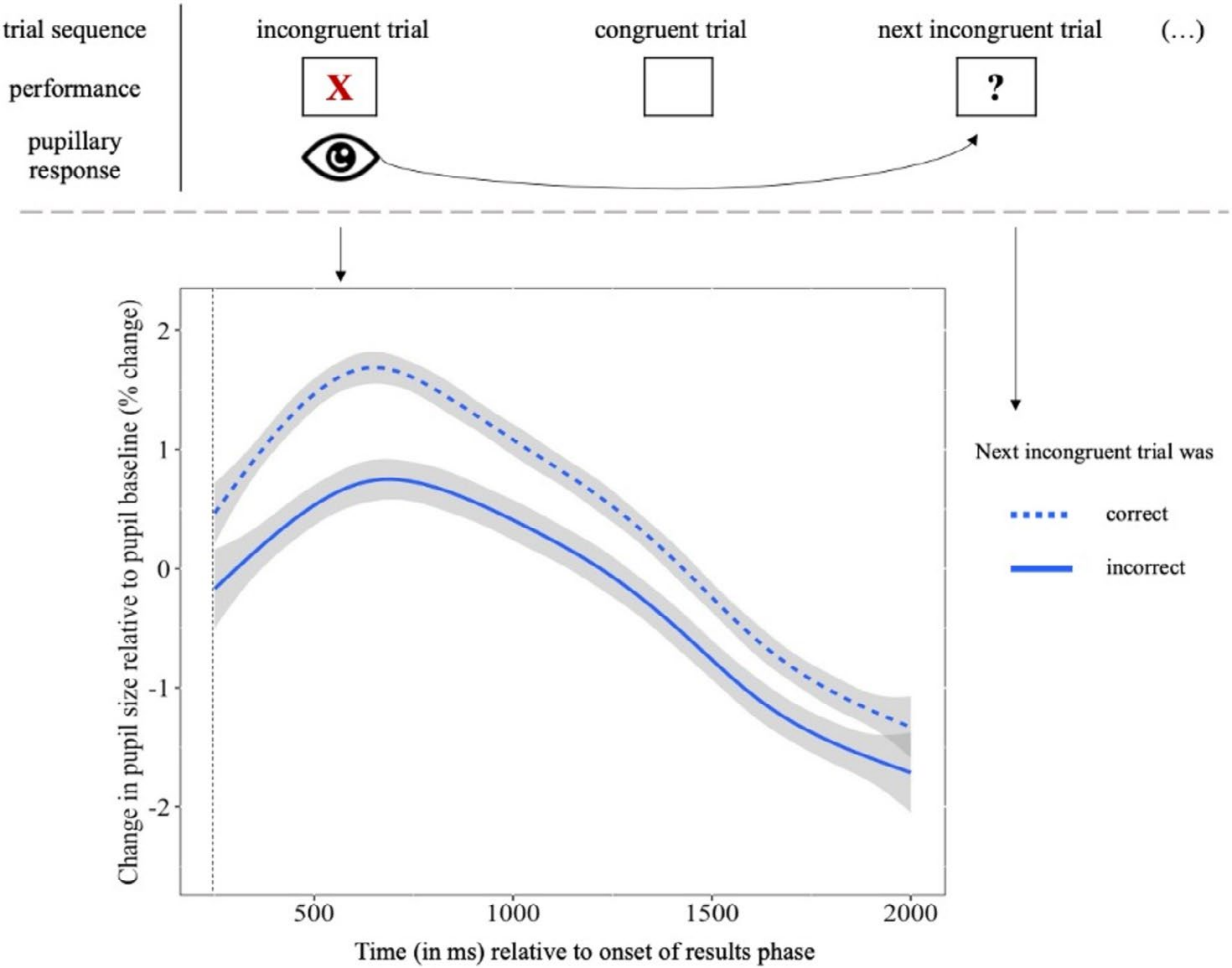
Pupil dilation response to incorrectly predicted, incongruent trials t as predictor of post‐error accuracy in trials t + 1. Incongruent trials that were predicted correctly were preceded by a larger pupil dilation response in the previous incongruent, incorrectly predicted trial (blue, dotted line) compared to incongruent trials that were predicted incorrectly (blue, solid line). Shading denotes *95% CI*. The dashed vertical line indicates the start of the time window of interest (250–2000 ms).

To test whether better conflict monitoring predicts belief revision, we tested whether the pupil dilation response to incorrectly predicted, incongruent trials predicted the likelihood of responding correctly to the next incongruent trial. Children who showed a greater pupil dilation response to incorrectly (vs. correctly) predicted, incongruent trials were more likely to switch to the correct concept on the next incongruent trial (*b* = −0.15, standardized *β* = −0.13, SE = 0.08, *p* = 0.048, see Figure 4). In other words, children who showed better conflict monitoring, as indexed by a stronger pupil dilation response, were more likely to subsequently revise their incorrect belief. Notably, this effect remained significant after controlling for age (*b* = −0.05, standardized *β* = −0.03, SE = 0.01, *p* = 0.047). This finding supports the hypothesis that conflict monitoring is important for subsequent belief revision throughout middle childhood.

### 4.4 Age Differences in Children's Belief Revision

**FIGURE 5:**
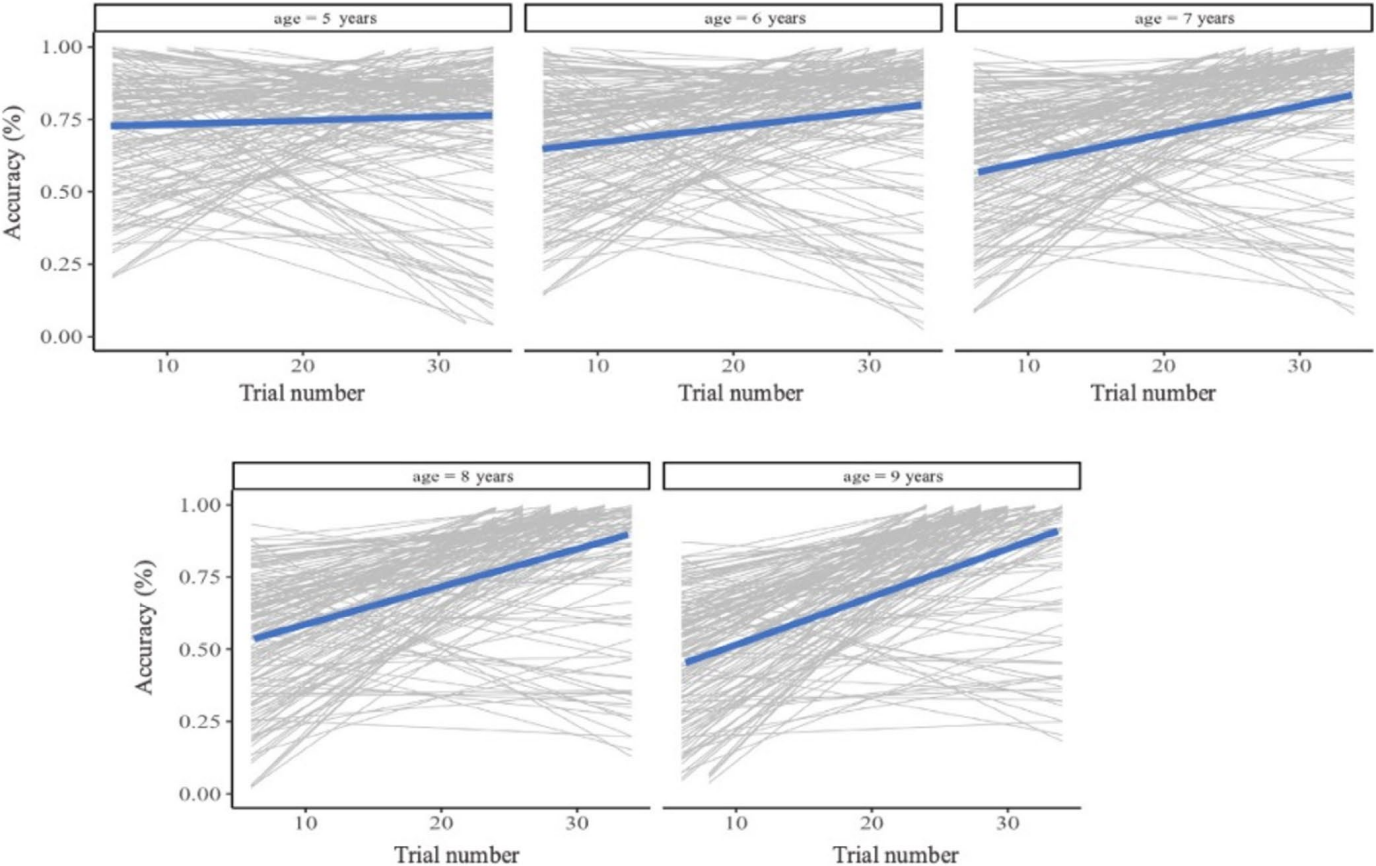
Percentage of correctly predicted, incongruent trials throughout the learning phase by age group. The gray lines represent each child's change in accuracy over the learning phase. The blue line represents the average change in children's accuracy over the learning phase for that age group.

Thus far, we have found that better conflict monitoring (operationalized by the pupillary surprise response) predicts subsequent belief revision. Building on the finding that conflict monitoring improves with age, we tested whether children would show faster belief revision with age during the learning phase, as indicated by a steeper performance increase on incongruent trials. First, we examined changes in children's performance across the learning phase. Regardless of age, we found that, on average, children improved over the learning phase (*b* = 0.01, standardized *β* = 0.16, SE < 0.01, *p* < 0.001, for details see Table S3, Model 1). However, we also found strong variability in learning (see Figure 5). A Levene test was not significant, *F*(3, 2693) = 2.16, *p* = 0.09, indicating homogeneity of variances across age groups. We then tested age as a moderator of average performance gains and found a steeper performance increase with age (*b* =  0.01, standardized *β* = 0.07, SE = 0.01, *p* = 0.008; see Table S3, Model 2 and Figure 5). Taken together, these results suggest that children revised their incorrect beliefs about water displacement more rapidly with age.

## 5 Discussion

The purpose of the present study was to examine the developmental relation between children's ability to monitor conflicts and their success in revising an intuitive theory. In a prediction–feedback task, we found that children's ability to monitor conflict in this task emerged around the ages of 6 to 7 and continued to improve across middle childhood. With increasing age, children showed slower response times when making an incorrect (vs. correct) prediction, indicating better pre‐conflict monitoring. Moreover, children showed a greater pupil dilation response when their predictions were incorrect, indicating better conflict monitoring. Better conflict monitoring, in turn, predicted a higher likelihood of subsequently revising an incorrect belief. In addition, children revised their misconception about water displacement more quickly with age, suggesting a role of conflict monitoring in the developmental progression of the ability to learn from incorrect predictions.

The results of this study are in line with developmental research on metacognitive monitoring, indicating that children demonstrate an increasing ability to monitor conflicts as they age. Metacognitive skills strongly improve during the early school years, evolving from basic to more sophisticated levels as children progress through formal education (Roebers 2014, 2017; Schneider and Löffler 2016). Recent research into cognitive development has shed light on the emergence of monitoring abilities during childhood, particularly during the crucial transition period around ages 6 to 7. For example, Roebers (2022) found age‐related boundaries in children's emerging ability to monitor cognitive conflict, with older children (7‐ and 8‐year‐olds) exhibiting the ability to monitor performance when compared to younger children (6‐year‐olds). In sum, metacognitive skills undergo significant development around the ages of 6 to 7, which could explain why conflict monitoring improves around these ages.

In addition, our results dovetail with a previous study by Ruggeri et al. (2019) that found that the efficacy of active learning emerges around age six and continues to develop throughout middle childhood. While not explicitly tested, this finding suggests that children's developing cognitive and metacognitive resources are a crucial aspect of realizing the benefits of active learning (Ruggeri et al. 2019). Our results suggest that metacognitive monitoring is one such skill that enables children to benefit from active learning activities.

In our study, both response times and the pupillary dilation response reflect these developmental patterns. Regarding the response time data, we found that with increasing age, children's response times to make an incorrect (vs. correct) prediction increased. Longer response times before making a prediction can validly indicate children's capacity to anticipate a conflict, as they have been shown to reflect diminished subjective confidence in an answer (Kälin and Roebers 2020; Koriat and Ackerman 2010). The response time data thus suggest that children's ability to anticipate a conflict improves with age.

Regarding the pupillary data, our results suggest that the magnitude of the pupillary dilation response can serve as a physiological indicator of enhanced conflict monitoring. Building on research showing that outcomes that conflict with one's expectations induce a pupillary dilation response (Braem et al. 2015; Brod et al. 2018; Theobald and Brod 2021), we found that children's pupil dilation response following incorrect (vs. correct) predictions became more pronounced with age, suggesting enhanced conflict monitoring. On a neural level, task‐evoked changes in pupil size are driven by the release of norepinephrine in the brainstem's locus coeruleus (Joshi et al. 2016), which receives input from the anterior cingulate cortex (ACC; Aston‐Jones and Cohen 2005). The ACC, whose functionality develops with age (Kelly et al. 2009), is thought to play a central role in conflict monitoring (Alexander and Brown 2019; Ebitz and Platt 2015; Metcalfe and Schwartz 2016). The ACC (as indexed by error‐related negativity) exhibits pronounced age‐related increases in activity in monitoring incorrect responses in 7‐ to 18‐year‐olds (Davies et al. 2004), suggesting that increased monitoring skills are associated with the development of the ACC. These neural developments thus correspond well with the age‐related development of children's conflict monitoring skills found in the present study. In other words, a possible explanation for why younger children might not show as pronounced a pupil dilation response in our task is that their conflict monitoring skills are still developing. Overall, the age‐related increase in the pupil dilation response to conflicting evidence suggests that children's conflict monitoring improves with age.

Monitoring a conflict or error is important for belief revision. In the present study, children who showed a larger pupil dilation response to an unexpected, theory‐incongruent trial were more likely to switch to the correct concept on the subsequent theory‐incongruent trial. Hence, children who showed enhanced conflict monitoring were more likely to revise their incorrect belief, suggesting that conflict monitoring benefited learning (Danielmeier and Ullsperger 2011). Our results are also consistent with research showing that a stronger pupil dilation response supports learning from errors. For example, the magnitude of the pupil dilation response following an error predicted a higher likelihood of being correct on the subsequent trial (Murphy et al. 2016; Theobald and Brod 2021). Thus, better conflict monitoring (as indexed by a greater pupil dilation response to incorrect predictions) may have initiated control processes that support the revision of incorrect beliefs.

This finding also aligns with existing research on science learning that underscores the role of conflict monitoring in promoting theory revision. According to newer versions of the “theory theory” of cognitive development (Gopnik and Wellman 2012), learners revise their theories rationally by testing hypotheses and revising their theory as new, conflicting data comes in. However, this presumes that learners accurately monitor when their hypothesis is proven incorrect. Therefore, conflict monitoring is essential to facilitate theory revision (Limón 2001; Limón and Carretero 1997). A recent study found that children who actively engaged in hypothesis testing (Colantonio et al. 2023b) showed better conflict monitoring, as indexed by a stronger pupil dilation response, and were more likely to subsequently revise their incorrect belief. These findings suggest that children who exhibited better conflict monitoring were better able to integrate empirical evidence into their existing intuitive frameworks, which, in turn, facilitated theory revision. Thus, our findings underline the focal role of conflict monitoring for the revision of intuitive theories and resonate with the core principles of “theory theory”.

Our findings that children's conflict monitoring skills, as indicated by slower response times and increased pupil dilation following incorrect predictions, predict faster belief revision align with research on metacognition in adults. For instance, Klaczynski (2006) found that in college students, learning occurred only when conditions activated metacognitive awareness, particularly in scenarios involving strong pre‐existing beliefs. Similarly, neuroscience studies have demonstrated that error monitoring processes in adults are crucial for correcting errors. For example, Butterfield and Mangels (2003) identified neural correlates of error detection and correction in a semantic retrieval task, showing that brain activity during error responses predicted subsequent error correction. Additionally, Yeung and Summerfield (2012) showed that neural activity during incorrect responses predicted future response accuracy, with error monitoring serving as a likely mechanism. These studies highlight the importance of conflict monitoring in guiding learning processes, a mechanism we observed even in young children.

In the present study, the age‐related improvements in conflict monitoring were correlated with faster belief revision throughout the learning task. As children's conflict monitoring skills develop with age, so too may their ability to benefit from hypothesis testing. Notably, the results held when controlling for study in the analyses (see Appendix C). Hence, it is unlikely that the differences in (pre)conflict monitoring or belief revision can be attributed to the differences in study design. These findings highlight the fundamental role of age‐related metacognitive skills in maximizing the benefits of learning from incorrect predictions. As children grow older, their metacognitive monitoring skills put them in a position to reap the benefits of discovery‐based, active learning activities (Brod 2021).

### 5.1 Limitations and Future Research

The results of this study should be viewed with some limitations that provide avenues for future research. First, age was measured in years rather than months. That is, a child who was almost 8 years old was in the same age category as a child who had just turned 7. For data protection reasons, we were not allowed to collect detailed personal data such as the date of birth of our participants, which unfortunately precluded a more fine‐grained analysis of age. A more exact assessment of age in months would have increased variation in age and may have better accounted for the continuous age trajectory. In addition, we used a cross‐sectional design to examine age‐related differences in children's conflict monitoring. Future studies could use a longitudinal design, testing the same children repeatedly (e.g., once a year), to elucidate the within‐person development of metacognitive monitoring skills across the elementary school years.

Second, future studies could use additional measures to examine the development of conflict monitoring skills. In the present study, we assessed children's conflict monitoring implicitly through their response times and pupil dilation responses. We used this implicit assessment because children at this age often struggle to explicitly report encountering a conflict (Theobald et al. 2024). However, for example, longer response times may also indicate higher item difficulty in general. Given the subjective nature of item difficulty—where children's theories (e.g., size or mass theories) affect how difficult they perceive a task to be—controlling for item difficulty comprehensively remains a challenge. A difficult item may encourage more thoughtful processing, which could reflect both difficulty and pre‐conflict monitoring simultaneously. Future studies could also explicitly ask children whether they recognized the conflict to investigate developmental differences in the ability to explicitly report conflict. Furthermore, in the present study, we assessed conflict monitoring in a specific task. Therefore, we cannot generalize good conflict monitoring in this particular task to other tasks. Future studies could assess children's conflict monitoring skills across multiple tasks to test whether children's conflict monitoring skills are task‐independent.

Future research could also test other moderators of children's conflict monitoring and belief revision. For example, it has been suggested that executive functions such as inhibition and task switching skills play an important role in conflict monitoring as well as belief revision (Mason and Zaccoletti 2021; Vosniadou 2019). Inhibition skills could help children to “stop and think” or check their prediction, as well as to suppress prior misconceptions (Roebers 2017). Switching skills could help children make comparisons, change their strategies, and consider different perspectives on a task (Vosniadou et al. 2018). As with metacognitive skills, inhibition and switching skills improve over the course of elementary school (Roebers 2017). Thus, examining the developmental trajectories of inhibition and switching and their role in belief revision seems a promising candidate for future research.

Contrary to literature on violation of expectancy, our results do not reveal a pupil dilation response to unexpected outcomes in younger children (5‐ and 6‐years‐old). This finding is in direct contrast with studies that have found that pupil dilation responses to unexpected outcomes occur even in infants (Zhang et al. 2018). Furthermore, Krüger et al. (2020) found that 1‐ to 6‐year‐olds showed pupillary surprise responses similar to that of adults in violation of expectancy paradigms. However, this difference could be due to the nature of our task. The task from Krürger and colleagues focused on perceptual processing (matching/mismatching stimuli) while our studies focused on more complex conceptual processing (experimental evidence). These differences in experimental tasks as well as analyses may contribute to the differences in (age‐related) pupil dilation response.

While our study highlights significant age‐related differences in conflict monitoring and belief revision, several alternative explanations warrant consideration. First, younger children's lack of a strong pupil response may reflect their less well‐formed beliefs about the task rather than their ability to recognize errors or surprise. However, this explanation is unlikely since the misconception that weight/mass plays a role in water displacement is a highly prevalent misconception even in young children (Dawson and Rowell 1984; Linn and Eylon 2000; Piaget and Inhelder 1941). Second, it is important to acknowledge that children's ability to remember their predictions may vary. We used a brief time interval between predictions and feedback (0.75–2 s), minimizing concerns related to memory retention. Still, while unlikely, younger children might face challenges in retaining their predictions, which could affect their conflict monitoring and belief revision. Future research should explore the extent to which memory for predictions influences conflict monitoring and belief revision across different age groups.

Finally, in this study, we synthesized data from three different studies to investigate the role of conflict monitoring in children's belief revision. While the key phases of the experiments were consistent across studies, which strengthens the reliability of our findings, it is important to acknowledge that differences in study design, participant engagement, or environmental factors across studies could potentially influence the results. Although we took measures to ensure methodological consistency, these factors cannot be completely ruled out as contributing to the age differences observed. Future research should aim to replicate these findings within a single study to further confirm the developmental nature of the results.

### 5.2 Practical Implications and Conclusion

The results of the present study highlight that the ability to identify conflicts between one's beliefs and data improves during the elementary school years, and that this has important implications for educational practice. For example, young children may have difficulty monitoring conflicting information accurately. The present study showed that conflict monitoring is important for revising erroneous beliefs. Therefore, if children do not monitor conflict, they may also have difficulty revising intuitive theories. Based on these findings, science teachers could use instructional methods to help children identify conflicts in their understanding, for example by providing reflection prompts (Theobald et al. 2024), and thereby support them in successfully revising their intuitive theories.

## Author Contributions

G.B., M.T., and L.S.W. designed the studies. G.B., E.R.H., M.T., and L.S.W. testing and data collection. E.R.H. and M.T. performed the data analyses. E.R.H. and M.T. performed the interpretation under the supervision of G.B. E.R.H. and M.T. drafted the paper. G.B. and L.S.W. provided critical revisions. All authors approved the final version of the paper for submission. E.R.H. and M.T. should be considered joint first authors.

## Conflicts of Interest

The authors declare no conflicts of interest.

## Supporting information


Data S1.

## Data Availability

The data and the analytic code necessary to reproduce the analyses as well as the materials are publicly available at the following URL: https://osf.io/bzsx8/. The analyses presented here were not preregistered.
