# Supplementary material for: Developing Conflict Monitoring Abilities Predict Children's Revision of an Intuitive Theory
Source: Child Dev. 2025 Apr 1;96(3):1207–19. doi: 10.1111/cdev.14241 (PMC12023835; doi:10.1111/cdev.14241)

**Appendix A**

**Detailed results tables**

**Supplementary Table 1**

*Prediction accuracy and age as predictors of response time to make prediction.*


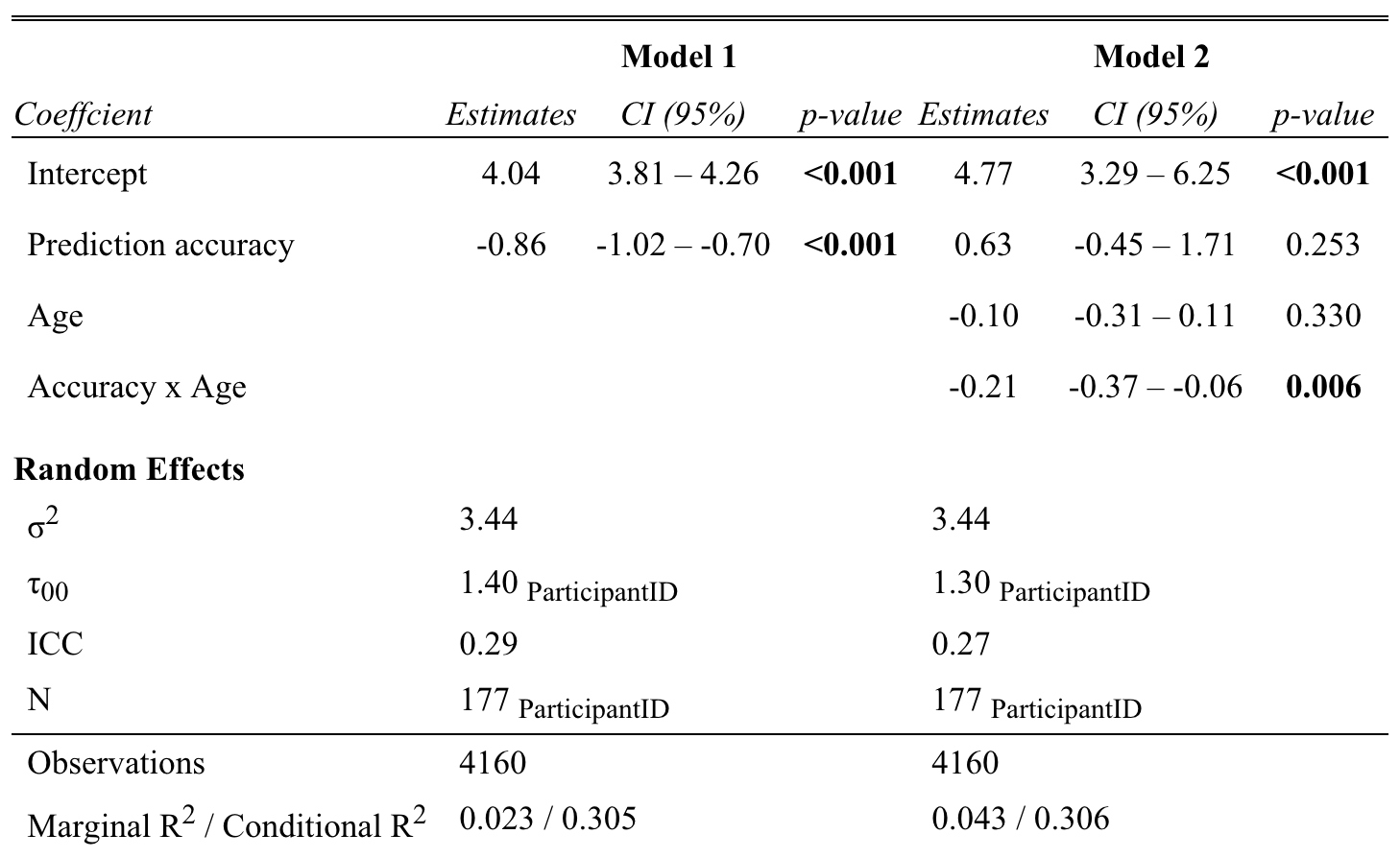


*Note.* Prediction accuracy was coded as follows: 0 = incorrect prediction, 1 = correct prediction.

**Supplementary Table 2**

*Prediction accuracy and age as predictors of pupil dilation response.*


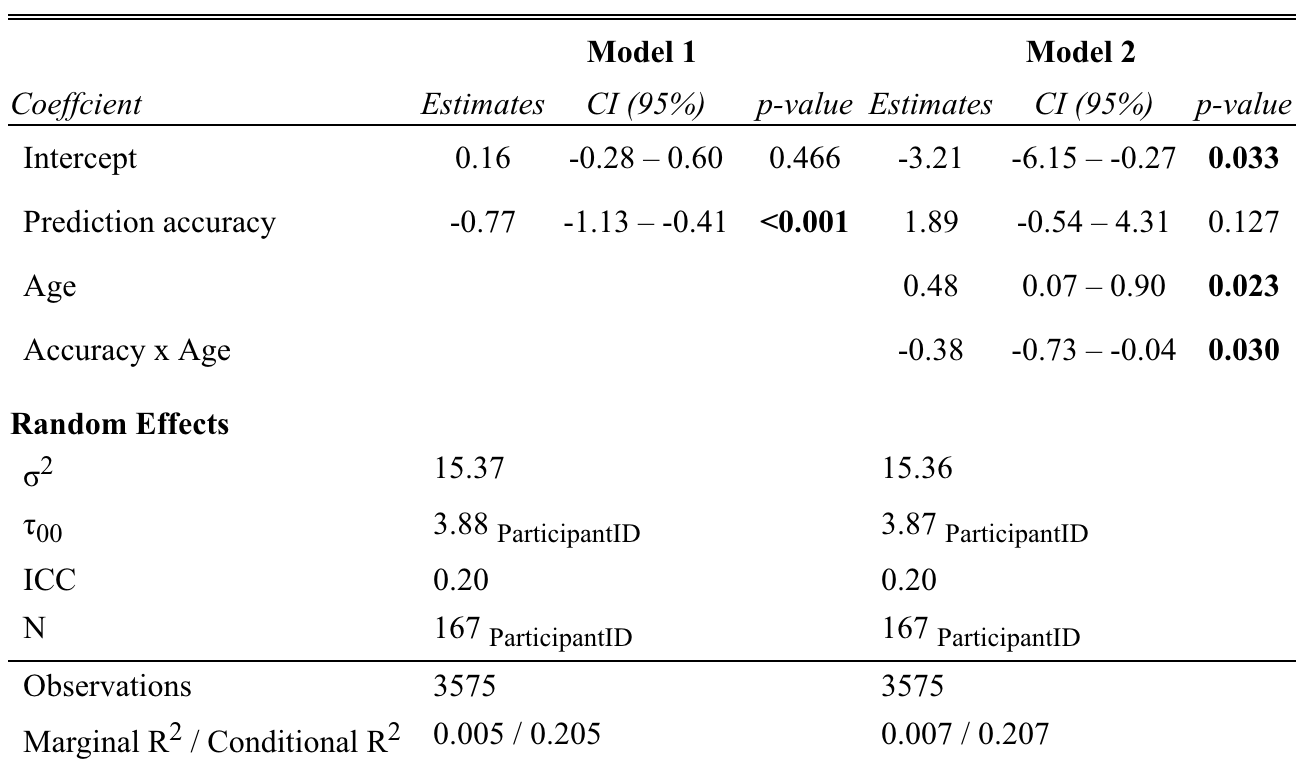


*Note.* Prediction accuracy was coded as follows: 0 = incorrect prediction, 1 = correct prediction.

**Supplementary Table 3**

*Trial number and age as predictors of prediction accuracy in incongruent trials.*


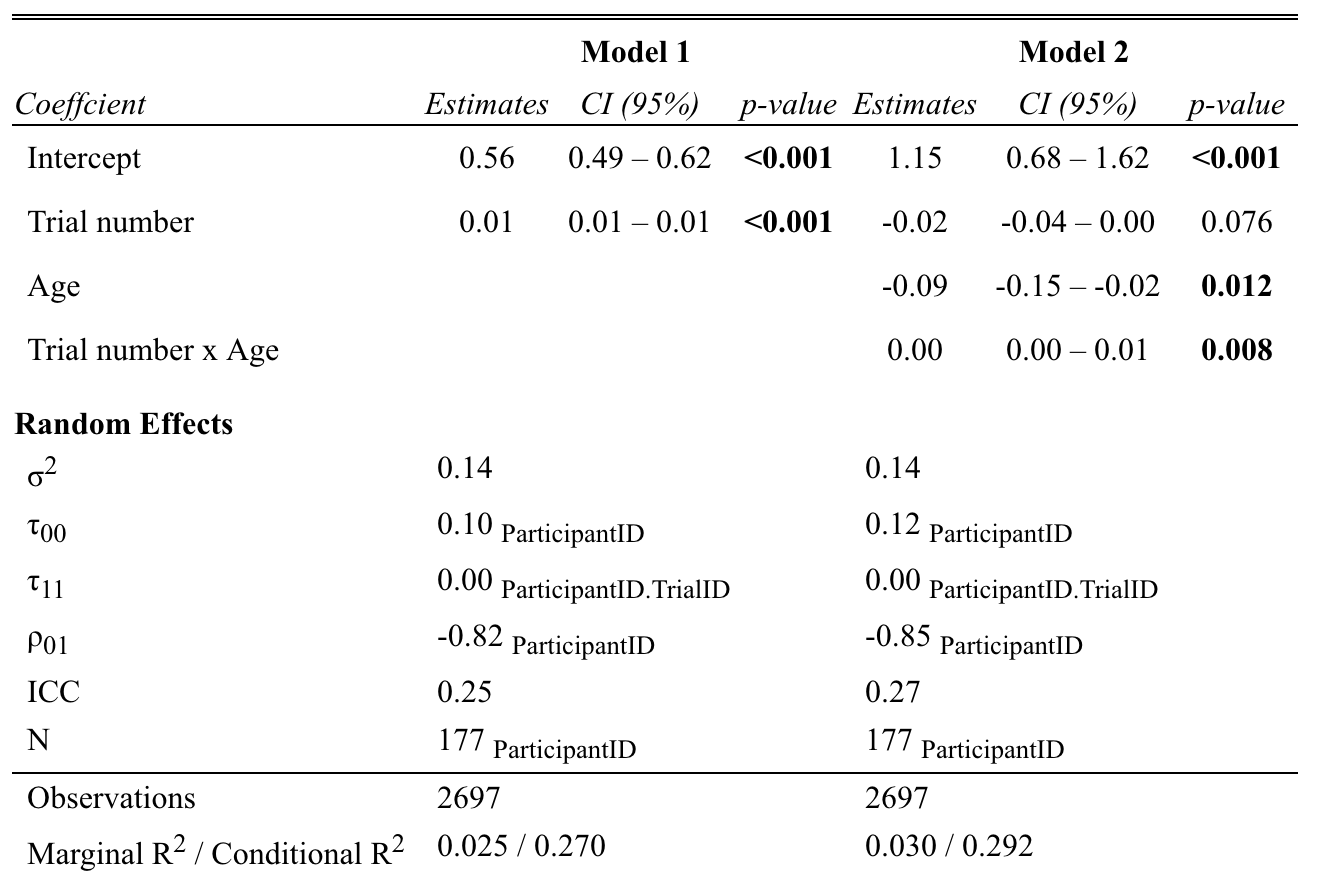


*Note.* Prediction accuracy was coded as follows: 0 = incorrect prediction, 1 = correct prediction.

**Appendix B**

Generalized additive mixed model

Generalized additive mixed models (GAMM) account for the variability of the pupil dilation signal via nonlinear random effects, the effect of gaze position on pupil dilation, and the auto-regression of the residuals. Therefore, we additionally calculated a GAMM on preprocessed pupil data (Van Rij et al., 2019). We used the R package “mgcv” (1.9-0; Wood, 2011) for modeling and “itsadug” (2.4.1; Van Rij et al., 2022) for visualization.

The purpose of this analysis was twofold. First, we used the analysis to generate the plots of the pupil dilation response to expected vs. unexpected trials (see Figure 3). Second, we aimed to test the robustness of our results reported in the main paper. However, for complex interactions (as tested in our paper), GAMMs are difficult to interpret (Van Rij et al., 2019). Therefore, we choose to report a hierarchical linear regression model in the main paper. However, for the sake of completeness, we report the results of the GAMM analysis below (see Supplementary Table 4).

We tested how well the GAMM that accounts for autocorrelation fits the data by examining the autocorrelation function, Q-Q plot, and residuals plotted against fitted values. Overall, we found that the autocorrelation of the residuals has been reduced by the GAMM procedure. Additionally, we tested how gaze position affected the pupil data. Including gaze position as a non-linear component was highly significant, suggesting that gaze position contributed significantly to explaining the variation in change in pupil size.

**Supplementary Table 4**

*Summary output of the GAMM model. The dependent variable was percent change in pupil size relative to baseline, and the independent variables were expectancy (unexpected, expected) and age (5, 6, 7, 8, 9).*

| *Coefficient* | *Estimates* | *Std. Error* | *p-value* |
| --- | --- | --- | --- |
| Intercept | -4.10 | 1.15 | **<.001** |
| Prediction accuracy | 2.64 | 1.32 | .046 |
| Age | 0.36 | 0.16 | **.026** |
| Accuracy x Age | -0.45 | 0.19 | **.017** |
| Observations: 197224 |  |  |  |
| R-sq. (adj): 0.988 |  |  |  |

**Appendix C**

Effect of study design

To ensure that the age differences observed in our findings were not due to variations in study design, we controlled for study in each of the models. Regarding pre-conflict monitoring, our analysis revealed a significant effect of study on response times for incorrect predictions (b = .70, standardized β = .23, SE = .16, *p* < .001). This suggests that study design contributed to differences in response time, likely due to procedural variations. Specifically, in Study 1, children were given a time limit to make their predictions, while in Studies 2 and 3, no such time limit was imposed. Despite this effect, the age-related difference in prediction accuracy remained significant after controlling for study (b = -.21, standardized β = -.04, SE = .08, *p* = .007), supporting the interpretation that pre-conflict monitoring improves with age (Supplementary Table 5). A significant effect of study on conflict monitoring, as indexed by pupil dilation, was also found (b = -.74, standardized β = -.13, SE = .31, *p* = .019). This could be attributed to differences in environmental factors across the studies, such as the fact that data collection occurred in different rooms due to unforeseen circumstances. However, the interaction between age and prediction accuracy remained significant (b = -.39, standardized β = -.04, SE = .18, *p* = .026), indicating that the age effect on conflict monitoring is robust, even when accounting for study differences (Supplementary Table 5). Importantly, there was no significant effect of study on age differences in belief revision, suggesting that the age-related improvements in belief revision were consistent across studies (Supplementary Table 6).

**Supplementary Table 5**

*Prediction accuracy and age as predictors of response time to make prediction (left) and of pupil dilation response (right) after controlling for study.*

| 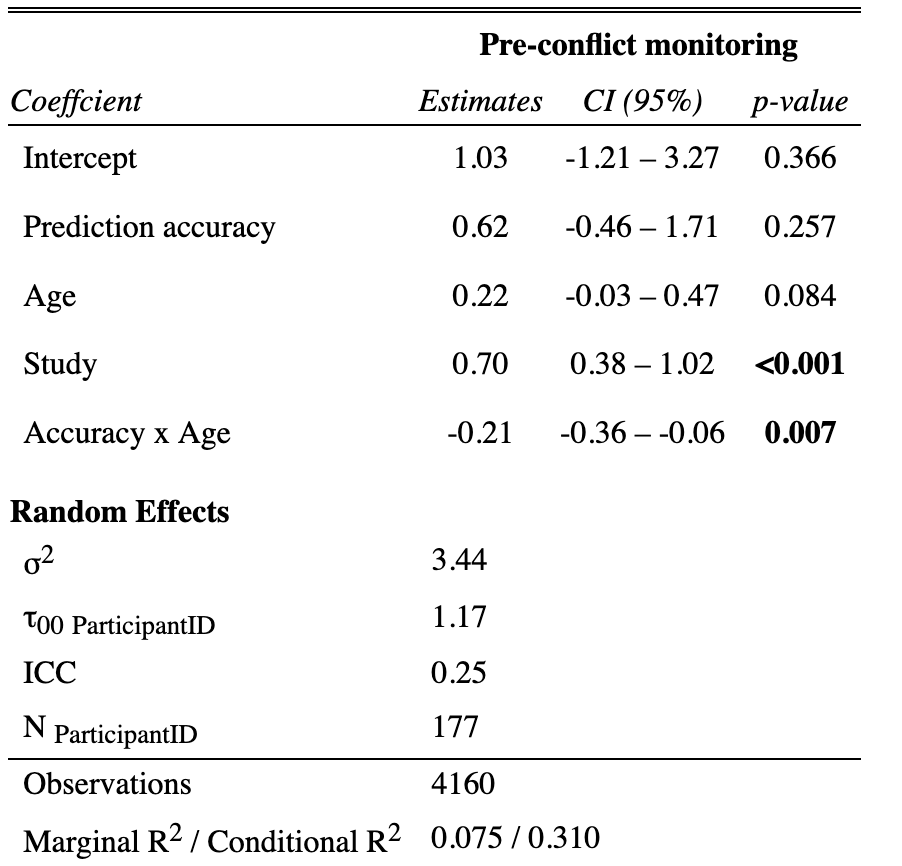 | 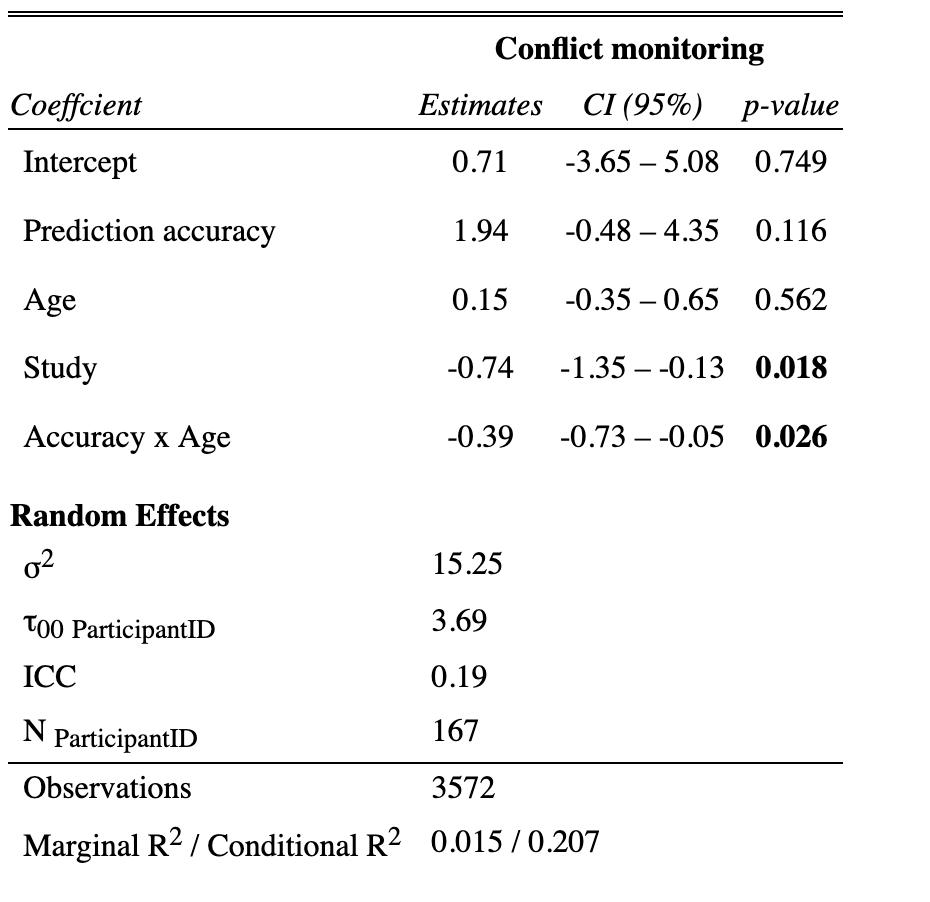 |
| --- | --- |

**Supplementary Table 6**

*Trial number and age as predictors of prediction accuracy in incongruent trials after controlling for study.*


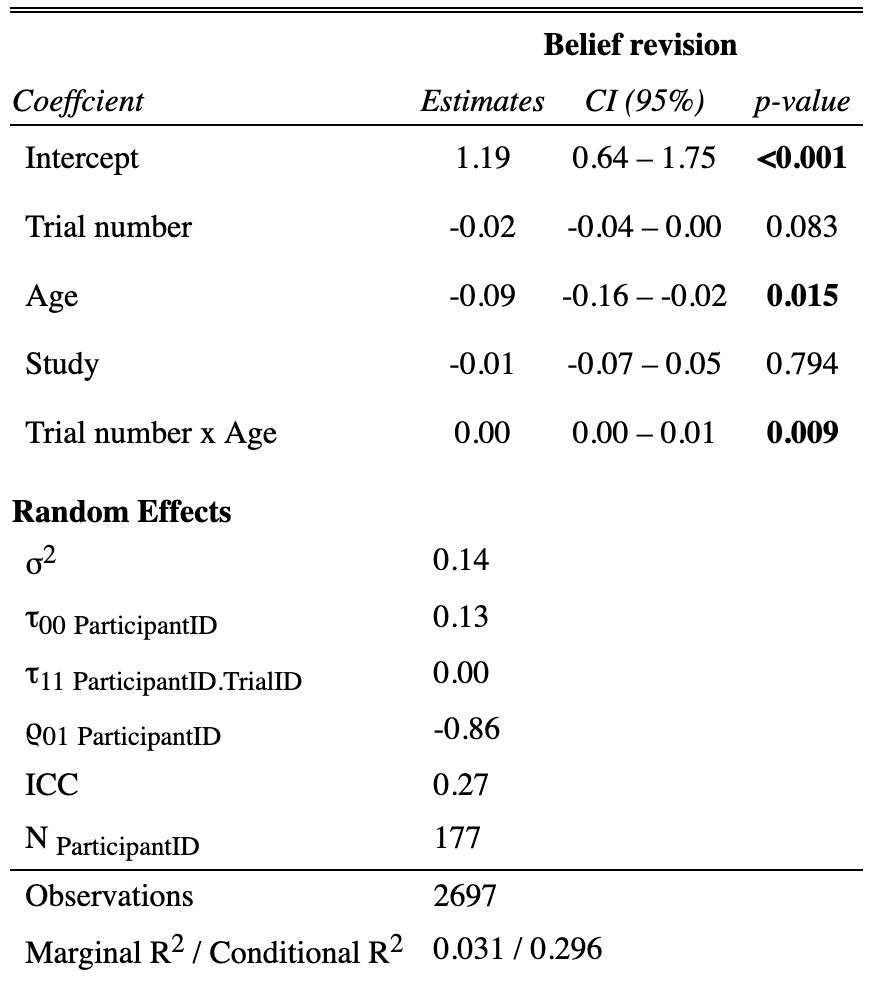

Supplement: Supplementary file 1 — Data S1. [file CDEV-96-1207-s001.docx]
